# 5-(4-Chloro­phen­yl)-7-(4-methyl­phen­yl)-4-(pyrrolidin-1-yl)-7*H*-pyrrolo­[2,3-*d*]pyrimidine

**DOI:** 10.1107/S1600536813019168

**Published:** 2013-07-20

**Authors:** Urmila H. Patel, Rajesh D. Modh, Dhaval A. Shah

**Affiliations:** a Department of Physics, Sardar Patel University, Vallabh Vidya Nagar, Gujarat 388 120, India; b Organic Syntheses Laboratory, M. G. Science Institute, Navarangpura, Ahmedabad 380 009, India

## Abstract

The title compound, C_23_H_21_ClN_4_, contains two molecules (*A* and *B*) in the asymmetric unit, which are related to one another by a pseudo-inversion center. The non-aromatic pyrrolidine ring in each independent mol­ecule adopts a half-chair conformation; the ring puckering parameters are θ = 0.407 (3) Å and ϕ = 270.5 (4)°, and the pseudo-rotation parameters are ρ = 72.5 (3)° and τ = 42.2 (2)° for an N—C bond of molecule *A*, and the corresponding values are 0.415 (3) Å, 271.6 (4)°, 73.6 (3)° and 42.6 (2)° for molecule *B*. The dihedral angles between the central fused-ring system and the substituted chlorophenyl and methylphenyl rings are 66.35 and 45.59°, respectively, for molecule *A*, and 64.51 and 41.89° for molecule *B*. The geometry of all four intramolecular C—H⋯π interactions are of type III. π–π interactions involving the centroids of symmetry-related pyrrole rings of molecule *B* are 4.390 Å, contributing further to the stability of the molecule.

## Related literature

For background to and the biological activity of pyrrolo­[2,3-*d*]pyrimidines, see: Chadwick (1990 ▶); Hulzenlaub *et al.* (1972 ▶); Ohgi *et al.* (1979 ▶); Smith *et al.* (1972 ▶). For our crystallographic investigations of heterocyclic compounds, see: Patel *et al.* (2007 ▶, 2012 ▶). For C—H⋯π inter­actions, see: Malone *et al.* (1997 ▶). For puckering parameters, see: Cremer & Pople (1975 ▶).
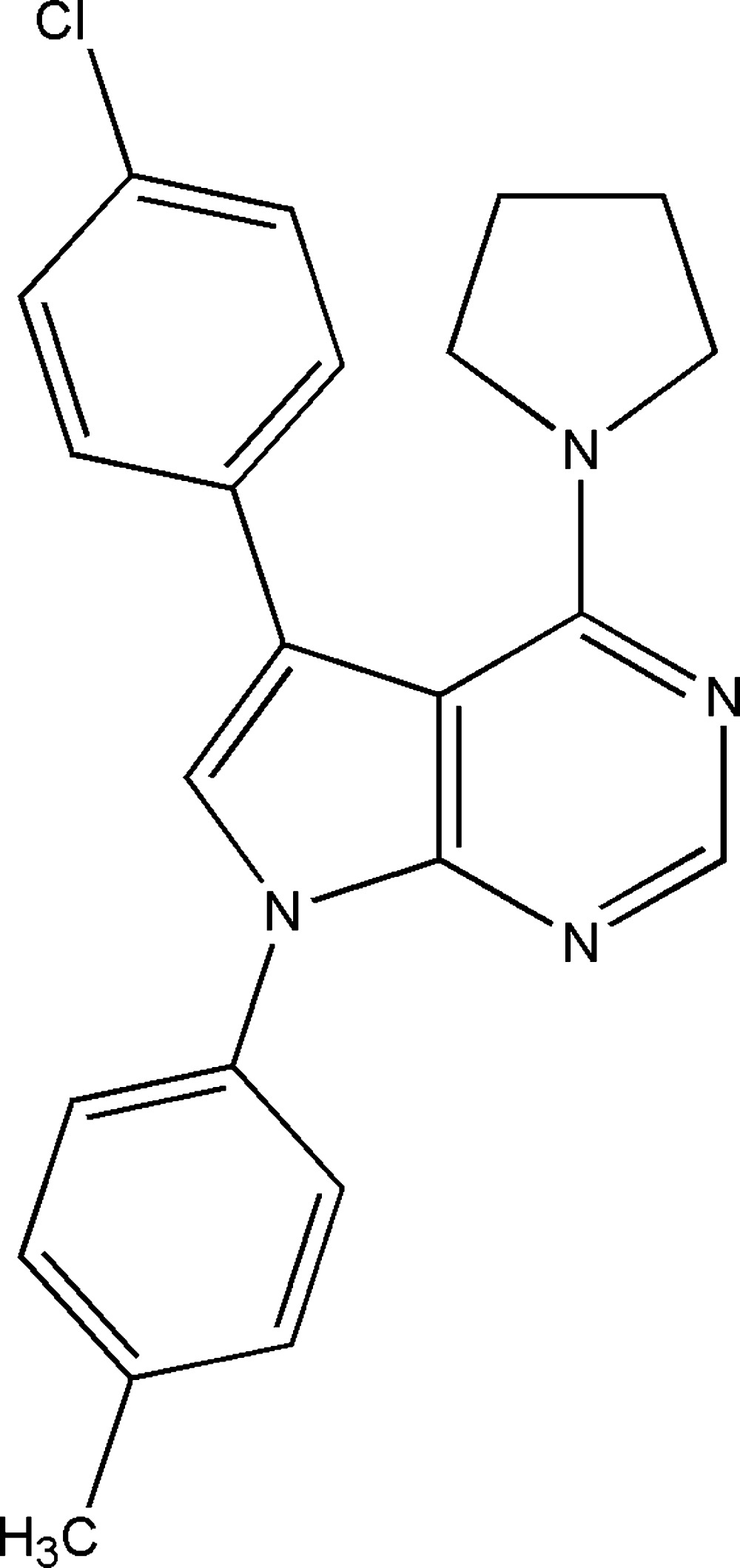


## Experimental

### 

#### Crystal data

C_23_H_21_ClN_4_
*M* *_r_* = 388.72
Triclinic, 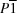

*a* = 8.967 (3) Å
*b* = 15.367 (5) Å
*c* = 15.960 (2) Å
α = 69.210 (17)°
β = 75.653 (16)°
γ = 76.52 (3)°
*V* = 1966.2 (10) Å^3^
*Z* = 4
Mo *K*α radiation
μ = 0.21 mm^−1^
*T* = 293 K
0.3 × 0.2 × 0.2 mm

#### Data collection

Enraf–Nonius CAD-4 diffractometer
Absorption correction: ψ scan (North *et al.*, 1968 ▶) *T*_min_ = 0.951, *T*_max_ = 0.959
7458 measured reflections
6864 independent reflections
3569 reflections with *I* > 2σ(*I*)
*R*_int_ = 0.027
2 standard reflections every 1 min intensity decay: none

#### Refinement

*R*[*F* ^2^ > 2σ(*F* ^2^)] = 0.042
*wR*(*F* ^2^) = 0.125
*S* = 1.01
6864 reflections
507 parameters
H-atom parameters constrained
Δρ_max_ = 0.18 e Å^−3^
Δρ_min_ = −0.25 e Å^−3^

### 

Data collection: *CAD-4 Software* (Enraf–Nonius, 1989 ▶); cell refinement: *CAD-4 Software*; data reduction: *CAD-4 Software*; program(s) used to solve structure: *SHELXS97* (Sheldrick, 2008 ▶); program(s) used to refine structure: *SHELXL97* (Sheldrick, 2008 ▶); molecular graphics: *PLATON* (Spek, 2009 ▶); software used to prepare material for publication: *publCIF* (Westrip, 2010 ▶).

## Supplementary Material

Crystal structure: contains datablock(s) global, I. DOI: 10.1107/S1600536813019168/gg2114sup1.cif

Structure factors: contains datablock(s) I. DOI: 10.1107/S1600536813019168/gg2114Isup2.hkl

Supplementary material file. DOI: 10.1107/S1600536813019168/gg2114Isup3.cml

Additional supplementary materials:  crystallographic information; 3D view; checkCIF report

## Figures and Tables

**Table 1:** Hydrogen-bond geometry (Å, °) *Cg*1, *Cg*2, *Cg*3 and *Cg*4 are the centroids of the N6/C5/C4/C9/N8/C7, N34/C33/C32/C37/N36/C35, C17–C22 and C45–C50 rings, respectively.

*D*—H⋯*A*	*D*—H	H⋯*A*	*D*⋯*A*	*D*—H⋯*A*
C18—H18⋯*Cg*1^i^	0.93	2.68	3.483 (3)	144
C46—H46⋯*Cg*2^ii^	0.93	2.73	3.549 (3)	147
C25—H251⋯*Cg*3	0.97	2.79	3.462 (3)	127
C53—H531⋯*Cg*4	0.97	2.84	3.506 (3)	127

Symmetry codes: (i) 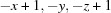; (ii) 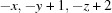.

**Fig. 1.**
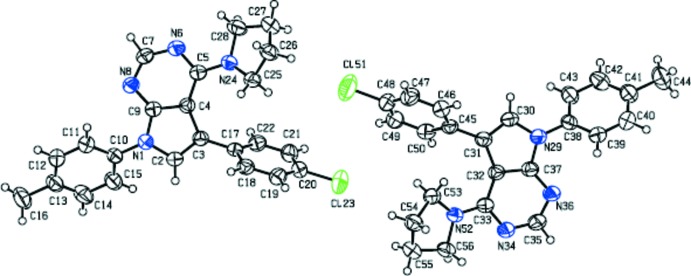
The molecular structure of (I), showing the atom-labelling scheme and 50% probability displacement ellipsoids.

**Fig. 2.**
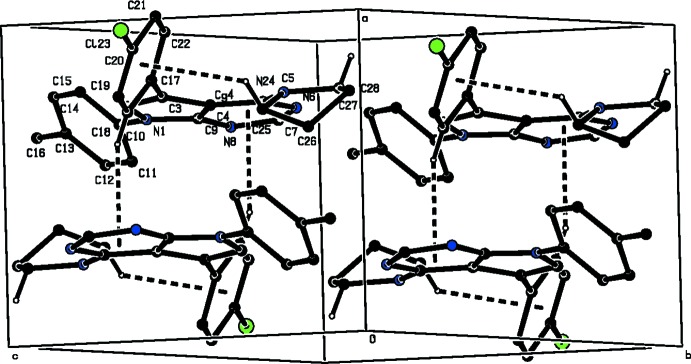
Part of the crystal structure showing the centrosymmetry related molecule A and molecule B which interlink by two pair of similar C—H···π hydrogen bond interaction, H-atoms have been omitted for clarity.

**Fig. 3.**
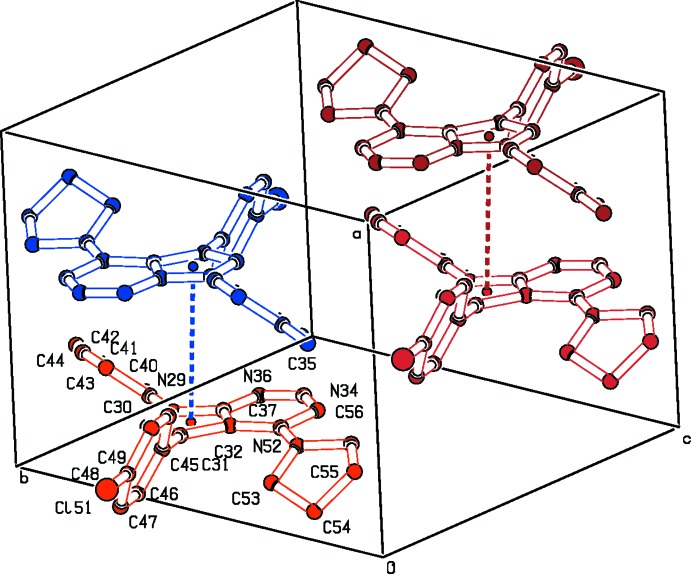
Part of the crystal structure Packing view of the molecule B along a axis showing the π–π interactions, H-atoms have been omitted for clarity.

## supplementary crystallographic information

### Comment

Pyrrolo[2,3-*d*]pyrimidine belongs to an important class of biologically active heterocyclic compounds, structurally closely related to nucleosides and some antibiotics (Chadwick, 1990; Ohgi *et al.*, 1979). These group of compounds are very well recognized for their biological activities such as anti-tumor, anti-allergic, anti-viral and anti-inflammatory (Hulzenlaub *et al.*, 1972; Smith *et al.*, 1972). As a part of a continuation of our crystallographic investigations of heterocyclic compounds (Patel *et al.*, 2007; Patel *et al.*, 2012), we now report herein the supra-molecular structure of a fused pyrrolo[2,3-*d*]pyrimidine derivative.

Two independent molecules A and B of the asymmetric unit [Fig.1] have similar conformations. The fused pyrrolo pyrimidine rings of A and B in the title compound are practically planar with a dihedral angle of 4.11 (16)° in A and 3.00 (17)° in B. The observed bond lengths and bond angles indicate a significant amount of strain arising due to fusion. A close observation of the molecular geometry: bond lengths, bond angles including torsional angles of both molecules A and B reveals that two independent molecules of the asymmetric unit are related to each other by a pseudoinversion center. The aromatic pyrrolodine ring of both molecules is puckered to adopt half chair conformation. The ring puckering parameters for mol A corresponding to the atom sequence N24—C25—C26—C27—C28 (Cremer *et al.*, 1975) are Theta = 0.407 (3)° and Phi = 270.5 (4)° and pseudo rotation parameters are Rho = 72.5 (3)° and t = 42.2 (2)° for N24—C25 bond and those of molecule B are Theta = 0.415 (3)° and Phi = 271.6 (4)° for the atom sequence N52—C53—C54—C55—C56 and the pseudo rotation parameters Rho = 73.6 (3)° and t = 42.6 (2)° for N52—C53 bond confirming a half chair conformation. The conformation of the substituents chlorophenyl, methylphenyl and pyrrolodine of both molecules are very similar. Torsional angles C4—C5—N24—C28 (177.9 (3)°) and C32—C33—N52—C53 (177.9 (3)°) confirm an extended conformation of the pyrrolodine ring with respect to fused ring system where as chlorophenyl ring of both molecules indicates a maximum turn, with dihedral angle of 66.35° (A)and 64.51°(B) between the said ring and fused system. The tolyl ring of both molecules are twisted by 44.59° and 41.89° for A and B, respectively.

In the absence of potential donor-acceptor groups in these heterocyclic compounds, the stability of supra-molecular structure is mainly due to relatively weak but significant C—H···π, π–π interactions. Molecule A and its centro-symmetry related pair gets superimposed centering at 0,0,1. The molecular aggregate so formed are held together by two pairs of C—H···π hydrogen bonds [Fig.2], one intra involving C25—H251 with *Cg*(3) (the centroid of the ring C17—C18—C19—C20—C21—C22) and the other intermolecular involving C18—H18 with *Cg*(1) (the centroid of the ring N6—C5—C4—C9—N8—C7) at 1-*x*, -*y*, 1-*z*. The C—H···π interactions involving molecule B and its symmetry related partner is very similar to that of molecule A but this time centered at 0,1,0. The intramolecular hydrogen bond involves C53—H531 to *Cg*(4) (the centroid of the ring C45—C46—C47—C48—C49—C50) and the intermolecular hydrogen bond is between C46—H46 with *Cg*(2) (the centroid of the ring N34—C33—C32—C37—N36—C35) at -*x*, 1 - *y*, 2 - *z*. The details of the geometry of these interation is in Table 1. The striking feature of the C—H···π hydrogen bond is that these interactions do not interlink molecules A and B, but it involves only individual molecules. The interactions involve the same group of moieties of the two molecules and is of same length. All of the four C—H···π interactions are of type-III as described by Malone *et al.*, 1997, indicating an exact similarities in the C—H···π interactions. In addition, direction specific π–π interaction involving symmetry related pyrrole ring of molecule B at -1-*x*, 1-*y*, 2-*z* contribute further to the stability of molecular packing along the [100] direction; their centroids are seperated by 4.390 Å [Fig.3]. However,this interaction is absent in molecule A. In the molecule, the closest approach distance between two symmetry (*x* - 1,-*y* + 1, *Z*+1) related chlorines is 3.883 (2) Å.

### Experimental

A uniform mixture of 4-chloro-5-(4-Chlorophenyl)-7-(4-methylphenyl)-7*H*- pyrrolo[2,3-*d*]pyrimidine and pyrrolodine was heated in an oil bath at 80–90° C with stirring. The heating was continued till the starting compound was consumed. The pH of reaction mixture was maintained and the final product was separated from a mixture of ethanol and *N*,*N*-dimethylformamide.

### Refinement

All the H atoms were placed in geometrically idealized positions with C—H distances of 0.96 Å (methyl) or 0.93 Å (aromatic) and constrained to ride on their parent atoms with *U*_iso_(H) = 1.2*U*_eq_(C) for the phenyl H atoms and *U*_iso_(H) = 1.5*U*_eq_(C) for the methyl H atoms.

### Crystal data

**Table:**

C_23_H_21_ClN_4_	*Z* = 4
*M**_r_* = 388.72	*F*(000) = 816
Triclinic, *P*1	*D*_x_ = 1.313 Mg m^−^^3^*D*_m_ = 1.310 Mg m^−^^3^*D*_m_ measured by floatation method
Hall symbol: -P 1	Melting point: 433 K
*a* = 8.967 (3) Å	Mo *K*α radiation, λ = 0.71073 Å
*b* = 15.367 (5) Å	Cell parameters from 25 reflections
*c* = 15.960 (2) Å	θ = 10–25°
α = 69.210 (17)°	µ = 0.21 mm^−^^1^
β = 75.653 (16)°	*T* = 293 K
γ = 76.52 (3)°	Needle, white
*V* = 1966.2 (10) Å^3^	0.3 × 0.2 × 0.2 mm

### Data collection

**Table:**

Enraf–Nonius CAD-4 diffractometer	3569 reflections with *I* > 2σ(*I*)
Radiation source: fine-focus sealed tube	*R*_int_ = 0.027
Graphite monochromator	θ_max_ = 25.0°, θ_min_ = 2.3°
ω–2θ scans	*h* = 0→10
Absorption correction: ψ scan (North *et al.*, 1968)	*k* = −17→18
*T*_min_ = 0.951, *T*_max_ = 0.959	*l* = −18→18
7458 measured reflections	2 standard reflections every 1 min
6909 independent reflections	intensity decay: none

### Refinement

**Table:**

Refinement on *F*^2^	Primary atom site location: structure-invariant direct methods
Least-squares matrix: full	Secondary atom site location: difference Fourier map
*R*[*F*^2^ > 2σ(*F*^2^)] = 0.042	Hydrogen site location: inferred from neighbouring sites
*wR*(*F*^2^) = 0.125	H-atom parameters constrained
*S* = 1.01	*w* = 1/[σ^2^(*F*_o_^2^) + (0.0544*P*)^2^ + 0.227*P*] where *P* = (*F*_o_^2^ + 2*F*_c_^2^)/3
6864 reflections	(Δ/σ)_max_ = 0.001
507 parameters	Δρ_max_ = 0.18 e Å^−^^3^
0 restraints	Δρ_min_ = −0.25 e Å^−^^3^

### Special details

**Table:**

Geometry. All esds (except the esd in the dihedral angle between two l.s. planes) are estimated using the full covariance matrix. The cell esds are taken into account individually in the estimation of esds in distances, angles and torsion angles; correlations between esds in cell parameters are only used when they are defined by crystal symmetry. An approximate (isotropic) treatment of cell esds is used for estimating esds involving l.s. planes.
Refinement. Refinement of *F*^2^ against ALL reflections. The weighted *R*-factor *wR* and goodness of fit *S* are based on *F*^2^, conventional *R*-factors *R* are based on *F*, with *F* set to zero for negative *F*^2^. The threshold expression of *F*^2^ > σ(*F*^2^) is used only for calculating *R*-factors(gt) *etc*. and is not relevant to the choice of reflections for refinement. *R*-factors based on *F*^2^ are statistically about twice as large as those based on *F*, and *R*- factors based on ALL data will be even larger.

### Fractional atomic coordinates and isotropic or equivalent isotropic displacement parameters (Å^2^)

**Table:**

	*x*	*y*	*z*	*U*_iso_*/*U*_eq_	
C2	0.2724 (3)	0.05271 (17)	0.38941 (16)	0.0460 (7)	
H2	0.2653	0.1114	0.3448	0.055*	
C3	0.2482 (3)	0.03931 (16)	0.48054 (16)	0.0411 (6)	
C4	0.2704 (3)	−0.06207 (16)	0.52460 (16)	0.0396 (6)	
C5	0.2610 (3)	−0.12783 (17)	0.61396 (17)	0.0446 (6)	
C7	0.3092 (3)	−0.24738 (18)	0.55056 (18)	0.0528 (7)	
H7	0.3204	−0.3120	0.5614	0.063*	
C9	0.3067 (3)	−0.10308 (17)	0.45445 (16)	0.0400 (6)	
C10	0.3198 (3)	−0.04409 (17)	0.28547 (16)	0.0440 (6)	
C11	0.4300 (3)	−0.11218 (19)	0.25792 (18)	0.0569 (8)	
H11	0.4996	−0.1513	0.2955	0.068*	
C12	0.4371 (4)	−0.12247 (19)	0.17456 (19)	0.0641 (8)	
H12	0.5110	−0.1694	0.1572	0.077*	
C13	0.3380 (4)	−0.0653 (2)	0.11640 (18)	0.0593 (8)	
C14	0.2295 (4)	0.0028 (2)	0.14461 (19)	0.0673 (9)	
H14	0.1615	0.0426	0.1063	0.081*	
C15	0.2187 (3)	0.0137 (2)	0.22876 (17)	0.0576 (8)	
H15	0.1434	0.0598	0.2467	0.069*	
C16	0.3471 (5)	−0.0769 (2)	0.02497 (19)	0.0868 (11)	
H161	0.4047	−0.1378	0.0243	0.130*	
H162	0.3986	−0.0287	−0.0223	0.130*	
H163	0.2437	−0.0716	0.0149	0.130*	
C17	0.2019 (3)	0.11753 (16)	0.52038 (15)	0.0385 (6)	
C18	0.3034 (3)	0.17926 (17)	0.50595 (17)	0.0469 (6)	
H18	0.4013	0.1724	0.4697	0.056*	
C19	0.2609 (4)	0.25081 (18)	0.54483 (18)	0.0547 (7)	
H19	0.3304	0.2911	0.5357	0.066*	
C20	0.1156 (4)	0.26198 (18)	0.59692 (17)	0.0526 (7)	
C21	0.0117 (3)	0.20315 (19)	0.61140 (18)	0.0542 (7)	
H21	−0.0868	0.2114	0.6466	0.065*	
C22	0.0553 (3)	0.13137 (17)	0.57289 (17)	0.0464 (6)	
H22	−0.0151	0.0915	0.5824	0.056*	
C25	0.2933 (3)	−0.02809 (17)	0.70083 (16)	0.0502 (7)	
H251	0.2101	0.0249	0.7018	0.060*	
H252	0.3776	−0.0073	0.6510	0.060*	
C26	0.3500 (3)	−0.06977 (19)	0.79062 (17)	0.0575 (8)	
H261	0.3441	−0.0215	0.8179	0.069*	
H262	0.4565	−0.1025	0.7832	0.069*	
C27	0.2377 (4)	−0.1378 (2)	0.84733 (17)	0.0616 (8)	
H271	0.2794	−0.1843	0.8990	0.074*	
H272	0.1375	−0.1048	0.8690	0.074*	
C28	0.2244 (4)	−0.1824 (2)	0.78017 (17)	0.0687 (9)	
H281	0.3079	−0.2351	0.7782	0.082*	
H282	0.1251	−0.2046	0.7959	0.082*	
C30	−0.2721 (3)	0.44162 (18)	1.08795 (17)	0.0534 (7)	
H30	−0.2631	0.3836	1.1335	0.064*	
C31	−0.2150 (3)	0.45403 (17)	0.99830 (17)	0.0468 (7)	
C32	−0.2535 (3)	0.55481 (16)	0.95198 (16)	0.0430 (6)	
C33	−0.2305 (3)	0.62018 (17)	0.86288 (17)	0.0458 (6)	
C35	−0.3802 (3)	0.73629 (19)	0.92152 (19)	0.0600 (8)	
H35	−0.4276	0.7991	0.9088	0.072*	
C37	−0.3365 (3)	0.59490 (17)	1.02000 (16)	0.0446 (6)	
C38	−0.4294 (3)	0.53417 (18)	1.18981 (16)	0.0468 (7)	
C39	−0.4229 (4)	0.60878 (19)	1.21646 (18)	0.0626 (8)	
H39	−0.3627	0.6546	1.1787	0.075*	
C40	−0.5069 (4)	0.6149 (2)	1.3000 (2)	0.0719 (9)	
H40	−0.5041	0.6664	1.3170	0.086*	
C41	−0.5943 (4)	0.5476 (2)	1.35894 (19)	0.0617 (8)	
C42	−0.5965 (3)	0.4730 (2)	1.33070 (19)	0.0637 (8)	
H42	−0.6538	0.4260	1.3693	0.076*	
C43	−0.5163 (3)	0.4657 (2)	1.24665 (17)	0.0552 (7)	
H43	−0.5211	0.4151	1.2289	0.066*	
C44	−0.6872 (4)	0.5557 (3)	1.4501 (2)	0.0919 (11)	
H441	−0.7890	0.5402	1.4592	0.138*	
H442	−0.6971	0.6191	1.4506	0.138*	
H443	−0.6342	0.5130	1.4981	0.138*	
C45	−0.1389 (3)	0.37392 (16)	0.96308 (16)	0.0426 (6)	
C46	−0.0005 (3)	0.32038 (18)	0.98691 (17)	0.0496 (7)	
H46	0.0462	0.3350	1.0251	0.060*	
C47	0.0700 (3)	0.24517 (19)	0.95481 (19)	0.0592 (8)	
H47	0.1640	0.2099	0.9708	0.071*	
C48	0.0001 (4)	0.22310 (19)	0.89926 (19)	0.0562 (7)	
C49	−0.1395 (4)	0.27394 (19)	0.87578 (18)	0.0563 (7)	
H49	−0.1871	0.2581	0.8388	0.068*	
C50	−0.2080 (3)	0.34898 (18)	0.90797 (17)	0.0508 (7)	
H50	−0.3026	0.3836	0.8924	0.061*	
C53	−0.0106 (3)	0.52431 (18)	0.78628 (17)	0.0521 (7)	
H531	−0.0438	0.4688	0.7858	0.062*	
H532	0.0390	0.5077	0.8386	0.062*	
C54	0.0984 (3)	0.56544 (19)	0.69815 (17)	0.0571 (7)	
H541	0.1655	0.6014	0.7071	0.069*	
H542	0.1625	0.5163	0.6742	0.069*	
C55	−0.0132 (3)	0.62861 (19)	0.63545 (17)	0.0574 (7)	
H551	−0.0603	0.5922	0.6133	0.069*	
H552	0.0386	0.6746	0.5840	0.069*	
C56	−0.1328 (3)	0.67548 (19)	0.69846 (16)	0.0576 (8)	
H561	−0.0987	0.7299	0.7008	0.069*	
H562	−0.2330	0.6952	0.6789	0.069*	
Cl23	0.06434 (12)	0.35242 (6)	0.64549 (6)	0.0915 (3)	
Cl51	0.08881 (13)	0.12936 (6)	0.85807 (7)	0.1017 (4)	
N1	0.3089 (2)	−0.03272 (14)	0.37193 (13)	0.0444 (5)	
N6	0.2761 (3)	−0.22063 (15)	0.62491 (14)	0.0556 (6)	
N8	0.3286 (2)	−0.19553 (14)	0.46364 (14)	0.0461 (5)	
N24	0.2366 (3)	−0.10608 (14)	0.69211 (13)	0.0507 (6)	
N29	−0.3456 (2)	0.52587 (14)	1.10356 (13)	0.0497 (6)	
N34	−0.3012 (3)	0.71100 (15)	0.84872 (15)	0.0586 (6)	
N36	−0.4013 (3)	0.68604 (15)	1.00855 (14)	0.0519 (6)	
N52	−0.1417 (3)	0.60092 (14)	0.78740 (13)	0.0492 (6)	

### Atomic displacement parameters (Å^2^)

**Table:**

	*U*^11^	*U*^22^	*U*^33^	*U*^12^	*U*^13^	*U*^23^
C2	0.0559 (17)	0.0360 (14)	0.0417 (16)	−0.0042 (12)	−0.0103 (13)	−0.0080 (12)
C3	0.0426 (15)	0.0390 (14)	0.0401 (15)	−0.0051 (12)	−0.0104 (12)	−0.0096 (12)
C4	0.0412 (15)	0.0386 (14)	0.0401 (14)	−0.0090 (11)	−0.0088 (11)	−0.0111 (12)
C5	0.0488 (17)	0.0395 (15)	0.0428 (15)	−0.0103 (12)	−0.0073 (12)	−0.0085 (12)
C7	0.070 (2)	0.0363 (15)	0.0526 (18)	−0.0150 (14)	−0.0114 (15)	−0.0106 (14)
C9	0.0386 (15)	0.0399 (15)	0.0418 (15)	−0.0072 (12)	−0.0102 (12)	−0.0105 (12)
C10	0.0522 (17)	0.0433 (15)	0.0341 (14)	−0.0117 (13)	−0.0034 (12)	−0.0099 (12)
C11	0.068 (2)	0.0485 (17)	0.0490 (17)	−0.0010 (15)	−0.0079 (15)	−0.0168 (14)
C12	0.089 (2)	0.0490 (18)	0.0499 (18)	−0.0106 (17)	0.0008 (17)	−0.0198 (15)
C13	0.079 (2)	0.0597 (19)	0.0394 (16)	−0.0269 (17)	0.0012 (15)	−0.0146 (15)
C14	0.075 (2)	0.080 (2)	0.0440 (17)	−0.0086 (18)	−0.0185 (15)	−0.0135 (16)
C15	0.0596 (19)	0.0644 (19)	0.0436 (16)	−0.0016 (15)	−0.0096 (14)	−0.0161 (14)
C16	0.131 (3)	0.093 (3)	0.0460 (18)	−0.041 (2)	−0.0030 (19)	−0.0286 (17)
C17	0.0438 (16)	0.0337 (13)	0.0340 (13)	−0.0030 (12)	−0.0123 (12)	−0.0044 (11)
C18	0.0463 (16)	0.0452 (15)	0.0438 (15)	−0.0098 (13)	−0.0031 (12)	−0.0094 (12)
C19	0.070 (2)	0.0444 (16)	0.0549 (17)	−0.0174 (15)	−0.0123 (16)	−0.0162 (14)
C20	0.068 (2)	0.0459 (16)	0.0412 (15)	−0.0020 (15)	−0.0100 (14)	−0.0153 (13)
C21	0.0524 (18)	0.0537 (17)	0.0496 (16)	0.0007 (15)	−0.0036 (14)	−0.0178 (14)
C22	0.0432 (16)	0.0434 (15)	0.0500 (16)	−0.0067 (12)	−0.0097 (13)	−0.0108 (13)
C25	0.0644 (19)	0.0439 (15)	0.0422 (15)	−0.0078 (13)	−0.0169 (13)	−0.0092 (12)
C26	0.072 (2)	0.0575 (17)	0.0426 (16)	−0.0152 (15)	−0.0180 (14)	−0.0070 (14)
C27	0.073 (2)	0.0655 (19)	0.0402 (16)	−0.0132 (16)	−0.0077 (15)	−0.0105 (14)
C28	0.104 (3)	0.0559 (18)	0.0418 (17)	−0.0307 (18)	−0.0082 (16)	−0.0021 (14)
C30	0.0649 (19)	0.0356 (15)	0.0475 (17)	−0.0055 (13)	−0.0038 (14)	−0.0043 (12)
C31	0.0496 (17)	0.0414 (15)	0.0419 (15)	−0.0075 (13)	−0.0030 (13)	−0.0074 (12)
C32	0.0404 (15)	0.0388 (14)	0.0436 (15)	−0.0018 (12)	−0.0061 (12)	−0.0096 (12)
C33	0.0493 (17)	0.0409 (15)	0.0430 (15)	−0.0017 (13)	−0.0141 (13)	−0.0081 (12)
C35	0.064 (2)	0.0447 (17)	0.0566 (19)	0.0113 (14)	−0.0131 (15)	−0.0095 (15)
C37	0.0409 (15)	0.0409 (15)	0.0438 (15)	−0.0028 (12)	−0.0064 (12)	−0.0068 (13)
C38	0.0500 (17)	0.0459 (16)	0.0360 (14)	0.0002 (13)	−0.0080 (13)	−0.0077 (12)
C39	0.085 (2)	0.0423 (16)	0.0510 (18)	−0.0099 (15)	−0.0010 (16)	−0.0106 (14)
C40	0.100 (3)	0.0532 (19)	0.061 (2)	−0.0037 (18)	−0.0095 (19)	−0.0247 (16)
C41	0.067 (2)	0.0632 (19)	0.0464 (17)	−0.0006 (16)	−0.0076 (15)	−0.0155 (15)
C42	0.063 (2)	0.074 (2)	0.0491 (18)	−0.0220 (17)	−0.0028 (15)	−0.0123 (16)
C43	0.0632 (19)	0.0609 (18)	0.0430 (16)	−0.0175 (15)	−0.0051 (14)	−0.0165 (14)
C44	0.103 (3)	0.106 (3)	0.058 (2)	0.001 (2)	−0.0004 (19)	−0.036 (2)
C45	0.0447 (16)	0.0350 (14)	0.0409 (14)	−0.0081 (12)	−0.0021 (12)	−0.0060 (11)
C46	0.0507 (17)	0.0481 (16)	0.0495 (16)	−0.0067 (14)	−0.0093 (13)	−0.0153 (13)
C47	0.0533 (18)	0.0490 (17)	0.0660 (19)	0.0018 (14)	−0.0098 (15)	−0.0140 (15)
C48	0.067 (2)	0.0441 (16)	0.0557 (17)	−0.0085 (15)	−0.0030 (15)	−0.0188 (14)
C49	0.069 (2)	0.0515 (17)	0.0519 (17)	−0.0158 (16)	−0.0115 (15)	−0.0165 (14)
C50	0.0498 (17)	0.0469 (16)	0.0502 (16)	−0.0064 (13)	−0.0124 (13)	−0.0073 (13)
C53	0.0548 (18)	0.0440 (15)	0.0471 (16)	−0.0037 (13)	−0.0021 (13)	−0.0097 (13)
C54	0.0581 (19)	0.0569 (17)	0.0463 (16)	−0.0085 (14)	−0.0030 (14)	−0.0090 (14)
C55	0.0619 (19)	0.0650 (19)	0.0410 (15)	−0.0142 (15)	−0.0069 (14)	−0.0107 (14)
C56	0.070 (2)	0.0535 (17)	0.0388 (15)	−0.0017 (15)	−0.0145 (14)	−0.0039 (13)
Cl23	0.1234 (8)	0.0718 (6)	0.0892 (6)	−0.0090 (5)	−0.0045 (6)	−0.0513 (5)
Cl51	0.1162 (8)	0.0781 (6)	0.1212 (8)	0.0104 (6)	−0.0169 (6)	−0.0636 (6)
N1	0.0531 (14)	0.0415 (12)	0.0384 (12)	−0.0056 (10)	−0.0099 (10)	−0.0124 (10)
N6	0.0789 (17)	0.0413 (13)	0.0460 (14)	−0.0189 (12)	−0.0083 (12)	−0.0091 (11)
N8	0.0534 (14)	0.0403 (12)	0.0467 (13)	−0.0121 (10)	−0.0101 (11)	−0.0126 (11)
N24	0.0726 (16)	0.0429 (13)	0.0360 (12)	−0.0179 (11)	−0.0061 (11)	−0.0087 (10)
N29	0.0565 (15)	0.0397 (12)	0.0412 (13)	−0.0012 (11)	−0.0010 (11)	−0.0083 (10)
N34	0.0714 (17)	0.0448 (14)	0.0460 (13)	0.0102 (12)	−0.0124 (12)	−0.0089 (11)
N36	0.0546 (15)	0.0433 (13)	0.0450 (14)	0.0066 (11)	−0.0098 (11)	−0.0075 (11)
N52	0.0589 (15)	0.0413 (12)	0.0367 (12)	0.0006 (11)	−0.0073 (11)	−0.0061 (10)

### Geometric parameters (Å, º)

**Table:**

C2—C3	1.363 (3)	C30—C31	1.355 (3)
C2—N1	1.387 (3)	C30—N29	1.387 (3)
C2—H2	0.9300	C30—H30	0.9300
C3—C4	1.452 (3)	C31—C32	1.459 (3)
C3—C17	1.483 (3)	C31—C45	1.485 (3)
C4—C9	1.407 (3)	C32—C37	1.404 (3)
C4—C5	1.421 (3)	C32—C33	1.418 (3)
C5—N6	1.352 (3)	C33—N34	1.355 (3)
C5—N24	1.357 (3)	C33—N52	1.357 (3)
C7—N8	1.323 (3)	C35—N36	1.318 (3)
C7—N6	1.334 (3)	C35—N34	1.337 (3)
C7—H7	0.9300	C35—H35	0.9300
C9—N8	1.347 (3)	C37—N36	1.351 (3)
C9—N1	1.375 (3)	C37—N29	1.376 (3)
C10—C15	1.375 (4)	C38—C39	1.374 (4)
C10—C11	1.376 (3)	C38—C43	1.375 (4)
C10—N1	1.429 (3)	C38—N29	1.430 (3)
C11—C12	1.379 (4)	C39—C40	1.383 (4)
C11—H11	0.9300	C39—H39	0.9300
C12—C13	1.374 (4)	C40—C41	1.375 (4)
C12—H12	0.9300	C40—H40	0.9300
C13—C14	1.374 (4)	C41—C42	1.378 (4)
C13—C16	1.512 (4)	C41—C44	1.519 (4)
C14—C15	1.389 (4)	C42—C43	1.386 (4)
C14—H14	0.9300	C42—H42	0.9300
C15—H15	0.9300	C43—H43	0.9300
C16—H161	0.9600	C44—H441	0.9600
C16—H162	0.9600	C44—H442	0.9600
C16—H163	0.9600	C44—H443	0.9600
C17—C22	1.387 (3)	C45—C46	1.377 (3)
C17—C18	1.388 (3)	C45—C50	1.387 (3)
C18—C19	1.382 (3)	C46—C47	1.385 (4)
C18—H18	0.9300	C46—H46	0.9300
C19—C20	1.370 (4)	C47—C48	1.372 (4)
C19—H19	0.9300	C47—H47	0.9300
C20—C21	1.370 (4)	C48—C49	1.374 (4)
C20—Cl23	1.742 (3)	C48—Cl51	1.737 (3)
C21—C22	1.381 (3)	C49—C50	1.380 (4)
C21—H21	0.9300	C49—H49	0.9300
C22—H22	0.9300	C50—H50	0.9300
C25—N24	1.467 (3)	C53—N52	1.458 (3)
C25—C26	1.510 (3)	C53—C54	1.520 (3)
C25—H251	0.9700	C53—H531	0.9700
C25—H252	0.9700	C53—H532	0.9700
C26—C27	1.514 (4)	C54—C55	1.516 (4)
C26—H261	0.9700	C54—H541	0.9700
C26—H262	0.9700	C54—H542	0.9700
C27—C28	1.503 (4)	C55—C56	1.517 (4)
C27—H271	0.9700	C55—H551	0.9700
C27—H272	0.9700	C55—H552	0.9700
C28—N24	1.475 (3)	C56—N52	1.472 (3)
C28—H281	0.9700	C56—H561	0.9700
C28—H282	0.9700	C56—H562	0.9700
			
C3—C2—N1	111.1 (2)	C33—C32—C31	139.3 (2)
C3—C2—H2	124.5	N34—C33—N52	114.8 (2)
N1—C2—H2	124.5	N34—C33—C32	119.2 (2)
C2—C3—C4	106.1 (2)	N52—C33—C32	126.0 (2)
C2—C3—C17	123.5 (2)	N36—C35—N34	130.0 (2)
C4—C3—C17	130.3 (2)	N36—C35—H35	115.0
C9—C4—C5	114.5 (2)	N34—C35—H35	115.0
C9—C4—C3	106.3 (2)	N36—C37—N29	123.3 (2)
C5—C4—C3	139.1 (2)	N36—C37—C32	127.2 (2)
N6—C5—N24	115.3 (2)	N29—C37—C32	109.4 (2)
N6—C5—C4	119.1 (2)	C39—C38—C43	120.1 (2)
N24—C5—C4	125.6 (2)	C39—C38—N29	120.9 (2)
N8—C7—N6	129.5 (2)	C43—C38—N29	119.0 (2)
N8—C7—H7	115.3	C38—C39—C40	119.2 (3)
N6—C7—H7	115.3	C38—C39—H39	120.4
N8—C9—N1	123.6 (2)	C40—C39—H39	120.4
N8—C9—C4	127.2 (2)	C41—C40—C39	122.4 (3)
N1—C9—C4	109.1 (2)	C41—C40—H40	118.8
C15—C10—C11	119.3 (2)	C39—C40—H40	118.8
C15—C10—N1	119.6 (2)	C40—C41—C42	116.9 (3)
C11—C10—N1	121.0 (2)	C40—C41—C44	121.8 (3)
C10—C11—C12	120.0 (3)	C42—C41—C44	121.3 (3)
C10—C11—H11	120.0	C41—C42—C43	122.2 (3)
C12—C11—H11	120.0	C41—C42—H42	118.9
C13—C12—C11	121.9 (3)	C43—C42—H42	118.9
C13—C12—H12	119.1	C38—C43—C42	119.2 (3)
C11—C12—H12	119.1	C38—C43—H43	120.4
C14—C13—C12	117.4 (3)	C42—C43—H43	120.4
C14—C13—C16	121.1 (3)	C41—C44—H441	109.5
C12—C13—C16	121.5 (3)	C41—C44—H442	109.5
C13—C14—C15	121.8 (3)	H441—C44—H442	109.5
C13—C14—H14	119.1	C41—C44—H443	109.5
C15—C14—H14	119.1	H441—C44—H443	109.5
C10—C15—C14	119.6 (3)	H442—C44—H443	109.5
C10—C15—H15	120.2	C46—C45—C50	118.2 (2)
C14—C15—H15	120.2	C46—C45—C31	120.8 (2)
C13—C16—H161	109.5	C50—C45—C31	120.9 (2)
C13—C16—H162	109.5	C45—C46—C47	121.0 (3)
H161—C16—H162	109.5	C45—C46—H46	119.5
C13—C16—H163	109.5	C47—C46—H46	119.5
H161—C16—H163	109.5	C48—C47—C46	119.5 (3)
H162—C16—H163	109.5	C48—C47—H47	120.3
C22—C17—C18	118.0 (2)	C46—C47—H47	120.3
C22—C17—C3	120.9 (2)	C47—C48—C49	121.0 (3)
C18—C17—C3	121.0 (2)	C47—C48—Cl51	119.7 (2)
C19—C18—C17	120.8 (2)	C49—C48—Cl51	119.3 (2)
C19—C18—H18	119.6	C48—C49—C50	118.9 (3)
C17—C18—H18	119.6	C48—C49—H49	120.6
C20—C19—C18	119.6 (3)	C50—C49—H49	120.6
C20—C19—H19	120.2	C49—C50—C45	121.5 (3)
C18—C19—H19	120.2	C49—C50—H50	119.3
C21—C20—C19	121.1 (3)	C45—C50—H50	119.3
C21—C20—Cl23	120.0 (2)	N52—C53—C54	103.2 (2)
C19—C20—Cl23	118.9 (2)	N52—C53—H531	111.1
C20—C21—C22	119.1 (3)	C54—C53—H531	111.1
C20—C21—H21	120.5	N52—C53—H532	111.1
C22—C21—H21	120.5	C54—C53—H532	111.1
C21—C22—C17	121.4 (3)	H531—C53—H532	109.1
C21—C22—H22	119.3	C55—C54—C53	102.9 (2)
C17—C22—H22	119.3	C55—C54—H541	111.2
N24—C25—C26	103.9 (2)	C53—C54—H541	111.2
N24—C25—H251	111.0	C55—C54—H542	111.2
C26—C25—H251	111.0	C53—C54—H542	111.2
N24—C25—H252	111.0	H541—C54—H542	109.1
C26—C25—H252	111.0	C54—C55—C56	101.6 (2)
H251—C25—H252	109.0	C54—C55—H551	111.5
C25—C26—C27	102.6 (2)	C56—C55—H551	111.5
C25—C26—H261	111.2	C54—C55—H552	111.5
C27—C26—H261	111.2	C56—C55—H552	111.5
C25—C26—H262	111.2	H551—C55—H552	109.3
C27—C26—H262	111.2	N52—C56—C55	103.4 (2)
H261—C26—H262	109.2	N52—C56—H561	111.1
C28—C27—C26	102.1 (2)	C55—C56—H561	111.1
C28—C27—H271	111.4	N52—C56—H562	111.1
C26—C27—H271	111.4	C55—C56—H562	111.1
C28—C27—H272	111.4	H561—C56—H562	109.0
C26—C27—H272	111.4	C9—N1—C2	107.42 (19)
H271—C27—H272	109.2	C9—N1—C10	126.5 (2)
N24—C28—C27	104.3 (2)	C2—N1—C10	125.3 (2)
N24—C28—H281	110.9	C7—N6—C5	118.3 (2)
C27—C28—H281	110.9	C7—N8—C9	111.1 (2)
N24—C28—H282	110.9	C5—N24—C25	124.8 (2)
C27—C28—H282	110.9	C5—N24—C28	119.1 (2)
H281—C28—H282	108.9	C25—N24—C28	109.5 (2)
C31—C30—N29	111.8 (2)	C37—N29—C30	106.9 (2)
C31—C30—H30	124.1	C37—N29—C38	128.0 (2)
N29—C30—H30	124.1	C30—N29—C38	124.7 (2)
C30—C31—C32	105.8 (2)	C35—N34—C33	117.7 (2)
C30—C31—C45	122.3 (2)	C35—N36—C37	110.8 (2)
C32—C31—C45	131.8 (2)	C33—N52—C53	125.8 (2)
C37—C32—C33	114.6 (2)	C33—N52—C56	119.9 (2)
C37—C32—C31	106.1 (2)	C53—N52—C56	110.9 (2)
			
N1—C2—C3—C4	−0.2 (3)	C30—C31—C45—C46	−65.2 (4)
N1—C2—C3—C17	−177.8 (2)	C32—C31—C45—C46	118.8 (3)
C2—C3—C4—C9	−0.2 (3)	C30—C31—C45—C50	112.5 (3)
C17—C3—C4—C9	177.2 (2)	C32—C31—C45—C50	−63.5 (4)
C2—C3—C4—C5	−177.6 (3)	C50—C45—C46—C47	1.7 (4)
C17—C3—C4—C5	−0.2 (5)	C31—C45—C46—C47	179.5 (2)
C9—C4—C5—N6	−4.5 (3)	C45—C46—C47—C48	−0.7 (4)
C3—C4—C5—N6	172.7 (3)	C46—C47—C48—C49	−0.6 (4)
C9—C4—C5—N24	176.0 (2)	C46—C47—C48—Cl51	179.5 (2)
C3—C4—C5—N24	−6.8 (5)	C47—C48—C49—C50	1.0 (4)
C5—C4—C9—N8	1.9 (4)	Cl51—C48—C49—C50	−179.2 (2)
C3—C4—C9—N8	−176.2 (2)	C48—C49—C50—C45	0.1 (4)
C5—C4—C9—N1	178.7 (2)	C46—C45—C50—C49	−1.4 (4)
C3—C4—C9—N1	0.6 (3)	C31—C45—C50—C49	−179.1 (2)
C15—C10—C11—C12	−0.6 (4)	N52—C53—C54—C55	−33.6 (3)
N1—C10—C11—C12	179.3 (2)	C53—C54—C55—C56	42.5 (3)
C10—C11—C12—C13	1.0 (4)	C54—C55—C56—N52	−34.9 (3)
C11—C12—C13—C14	−0.5 (4)	N8—C9—N1—C2	176.2 (2)
C11—C12—C13—C16	179.8 (3)	C4—C9—N1—C2	−0.7 (3)
C12—C13—C14—C15	−0.4 (4)	N8—C9—N1—C10	6.1 (4)
C16—C13—C14—C15	179.3 (3)	C4—C9—N1—C10	−170.8 (2)
C11—C10—C15—C14	−0.3 (4)	C3—C2—N1—C9	0.6 (3)
N1—C10—C15—C14	179.8 (2)	C3—C2—N1—C10	170.8 (2)
C13—C14—C15—C10	0.8 (5)	C15—C10—N1—C9	131.9 (3)
C2—C3—C17—C22	112.0 (3)	C11—C10—N1—C9	−48.0 (4)
C4—C3—C17—C22	−65.1 (4)	C15—C10—N1—C2	−36.5 (4)
C2—C3—C17—C18	−67.7 (3)	C11—C10—N1—C2	143.6 (3)
C4—C3—C17—C18	115.2 (3)	N8—C7—N6—C5	−1.2 (4)
C22—C17—C18—C19	1.7 (4)	N24—C5—N6—C7	−176.2 (2)
C3—C17—C18—C19	−178.6 (2)	C4—C5—N6—C7	4.3 (4)
C17—C18—C19—C20	−1.2 (4)	N6—C7—N8—C9	−1.5 (4)
C18—C19—C20—C21	0.2 (4)	N1—C9—N8—C7	−175.4 (2)
C18—C19—C20—Cl23	179.88 (19)	C4—C9—N8—C7	0.9 (4)
C19—C20—C21—C22	0.3 (4)	N6—C5—N24—C25	146.9 (2)
Cl23—C20—C21—C22	−179.42 (19)	C4—C5—N24—C25	−33.6 (4)
C20—C21—C22—C17	0.3 (4)	N6—C5—N24—C28	−1.6 (4)
C18—C17—C22—C21	−1.2 (4)	C4—C5—N24—C28	177.9 (3)
C3—C17—C22—C21	179.1 (2)	C26—C25—N24—C5	−138.3 (3)
N24—C25—C26—C27	−33.8 (3)	C26—C25—N24—C28	12.8 (3)
C25—C26—C27—C28	42.1 (3)	C27—C28—N24—C5	166.5 (2)
C26—C27—C28—N24	−34.2 (3)	C27—C28—N24—C25	13.6 (3)
N29—C30—C31—C32	0.5 (3)	N36—C37—N29—C30	177.5 (2)
N29—C30—C31—C45	−176.5 (2)	C32—C37—N29—C30	−1.7 (3)
C30—C31—C32—C37	−1.4 (3)	N36—C37—N29—C38	4.5 (4)
C45—C31—C32—C37	175.1 (3)	C32—C37—N29—C38	−174.7 (2)
C30—C31—C32—C33	177.5 (3)	C31—C30—N29—C37	0.7 (3)
C45—C31—C32—C33	−5.9 (5)	C31—C30—N29—C38	174.0 (2)
C37—C32—C33—N34	−6.7 (4)	C39—C38—N29—C37	−45.6 (4)
C31—C32—C33—N34	174.3 (3)	C43—C38—N29—C37	135.0 (3)
C37—C32—C33—N52	173.1 (2)	C39—C38—N29—C30	142.5 (3)
C31—C32—C33—N52	−5.8 (5)	C43—C38—N29—C30	−36.9 (4)
C33—C32—C37—N36	3.6 (4)	N36—C35—N34—C33	0.5 (5)
C31—C32—C37—N36	−177.2 (2)	N52—C33—N34—C35	−174.7 (3)
C33—C32—C37—N29	−177.4 (2)	C32—C33—N34—C35	5.1 (4)
C31—C32—C37—N29	1.9 (3)	N34—C35—N36—C37	−3.7 (4)
C43—C38—C39—C40	−1.2 (4)	N29—C37—N36—C35	−177.6 (3)
N29—C38—C39—C40	179.4 (3)	C32—C37—N36—C35	1.3 (4)
C38—C39—C40—C41	1.6 (5)	N34—C33—N52—C53	155.2 (2)
C39—C40—C41—C42	−0.7 (5)	C32—C33—N52—C53	−24.7 (4)
C39—C40—C41—C44	−179.4 (3)	N34—C33—N52—C56	−2.2 (4)
C40—C41—C42—C43	−0.6 (5)	C32—C33—N52—C56	177.9 (3)
C44—C41—C42—C43	178.1 (3)	C54—C53—N52—C33	−147.2 (3)
C39—C38—C43—C42	0.0 (4)	C54—C53—N52—C56	11.9 (3)
N29—C38—C43—C42	179.4 (2)	C55—C56—N52—C33	175.1 (2)
C41—C42—C43—C38	1.0 (4)	C55—C56—N52—C53	14.6 (3)

### Hydrogen-bond geometry (Å, º)

Cg1, Cg2, Cg3 and Cg4 are the centroids of the N6/C5/C4/C9/N8/C7, N34/C33/C32/C37/N36/C35, C17–C22 and C45–C50 rings, respectively.

**Table:**

*D*—H···*A*	*D*—H	H···*A*	*D*···*A*	*D*—H···*A*
C18—H18···*Cg*1^i^	0.93	2.68	3.483 (3)	144
C46—H46···*Cg*2^ii^	0.93	2.73	3.549 (3)	147
C25—H251···*Cg*3	0.97	2.79	3.462 (3)	127
C53—H531···*Cg*4	0.97	2.84	3.506 (3)	127

Symmetry codes: (i) −*x*+1, −*y*, −*z*+1; (ii) −*x*, −*y*+1, −*z*+2.

## References

[bb1] Chadwick, D. J. (1990). *The Chemistry of Heterocyclic Compounds*, Vol. 48, edited by R. A. Jones, pp. 1–104. New York: Willey & Sons.

[bb2] Cremer, D. & Pople, J. A. (1975). *J. Am. Chem. Soc.* **97**, 1354–1358.

[bb3] Enraf–Nonius (1989). *CAD-4 Software* Enraf–Nonius, Delft, The Netherlands.

[bb4] Hulzenlaub, W., Tolman, R. L. & Robins, R. K. (1972). *J. Med. Chem.* **15**, 879–883. 10.1021/jm00279a001 5051002

[bb5] Malone, J. F., Murray, C. M., Charlton, M. H., Docherty, R. & Lavery, A. J. (1997). *J. Chem. Soc. Faraday Trans.* **93**, 3429–3436.

[bb6] North, A. C. T., Phillips, D. C. & Mathews, F. S. (1968). *Acta Cryst.* A**24**, 351–359.

[bb7] Ohgi, T., Kondo, T. & Goto, T. (1979). *J. Am. Chem. Soc.* **101**, 3629–3633.

[bb8] Patel, U. H., Gandhi, S. A., Barot, V. M. & Patel, M. C. (2012). *Acta Cryst.* E**68**, o2926–o2927. 10.1107/S1600536812038275 PMC3470302 23125715

[bb9] Patel, U. H., Patel, P. D. & Thakker, N. (2007). *Acta Cryst.* C**63**, o337–o339. 10.1107/S0108270107017155 17551197

[bb10] Sheldrick, G. M. (2008). *Acta Cryst.* A**64**, 112–122. 10.1107/S0108767307043930 18156677

[bb11] Smith, C. W., Sidwell, R. W., Robins, R. K. & Tolman, R. L. (1972). *J. Med. Chem.* **15**, 883–887. 10.1021/jm00279a002 5065785

[bb12] Spek, A. L. (2009). *Acta Cryst.* D**65**, 148–155. 10.1107/S090744490804362X PMC2631630 19171970

[bb13] Westrip, S. P. (2010). *J. Appl. Cryst.* **43**, 920–925.

